# Supplementary material for: Context-dependent limb movement encoding in neuronal populations of motor cortex
Source: Nat Commun. 2019 Oct 23;10:4812. doi: 10.1038/s41467-019-12670-z (PMC6811620; doi:10.1038/s41467-019-12670-z)
Supplement: Supplementary file 1 — Supplementary Information [file 41467_2019_12670_MOESM1_ESM.pdf]

Supplementary Information

Context-dependent limb movement encoding in neuronal populations of motor cortex

Wolfgang Omlor, Anna-Sophia Wahl, Pia Sipilä, Henry Lütcke, Balazs Laurenczy, I-Wen Chen, Lazar T. Sumanovski, Marcel van 't Hoff, Philipp Bethge, Fabian F. Voigt, Martin E. Schwab, Fritjof Helmchen

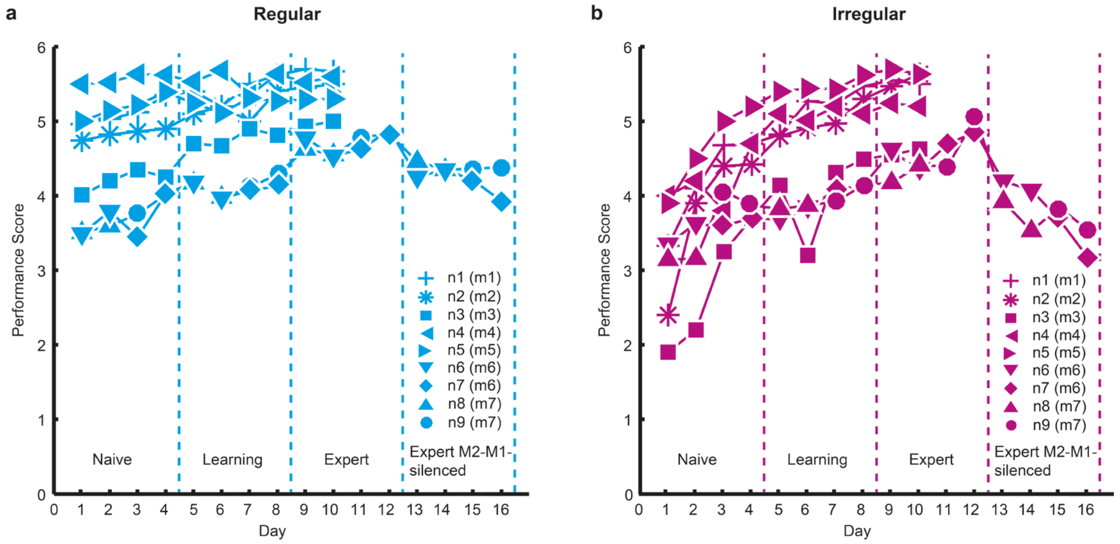

Supplementary Figure 1. Forelimb performance score during motor learning.
(**a**) Evolution of the forelimb performance score for individual mice (n = 7) during learning of skilled locomotion and silencing of M2-M1-projections on the regular pattern (cyan). m1 – m7 refers to mouse 1 to 7, n1-n9 to neuronal network 1 to 9; (**b**) Evolution of the forelimb performance score for the same mice during learning of skilled locomotion and silencing of M2-M1-projections on the irregular pattern (magenta); based on the performance score we divided motor learning during both conditions into ‘naive’ (days 1-4), ‘learning’ (days 5-8) and ‘expert’ phase (days 9-12, performance score at saturating level); in mice 1 to 5 (1st subset of experiments), calcium imaging was only applied during the expert phase; in mice 6 (n6 and n7) and 7 (n8 and n9), which correspond to the 2nd subset of experiments, we included an additional expert phase with silencing of M2-M1 projections (‘Expert M2-M1-silenced’), and calcium imaging was performed during all phases. Rating according to the forelimb performance score: 0 = Total miss; 1 = Deep slip; 2 = Slight slip; 3 = Replacement; 4 = Correction; 5 = Partial placement; 6 = Correct placement.

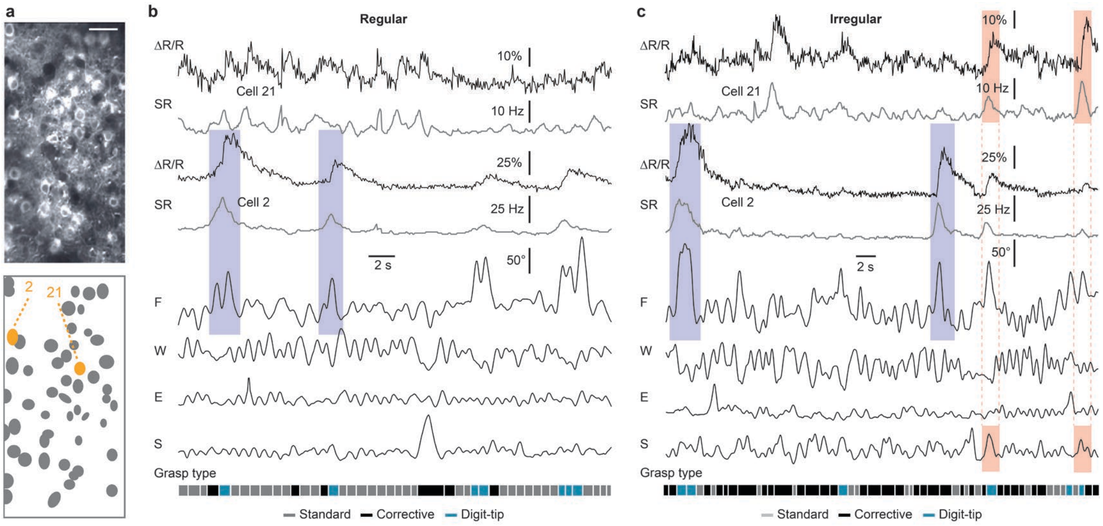

Supplementary Figure 2: Calcium imaging data and movement variables.
(**a**) Upper panel: Two-photon image of the YC-Nano140-expressing neuronal population that was measured with calcium imaging during skilled locomotion. Lower panel: Schematic of the selected regions of interest, with neurons 2 and 21 marked in orange. (**b**) Regular condition: Raw ΔR/R calcium signals (black traces) and deconvolved spiking rates (SR, dark grey traces) for the two cells marked in a, along with simultaneously recorded joint angles and the classified grasp types. (**c**) Irregular condition: Same conventions as in (b). Salient neuronal responses to finger base movements are indicated by the blue shaded areas. Salient neuronal responses to shoulder movements are highlighted by red shaded areas. Data from mouse 1.

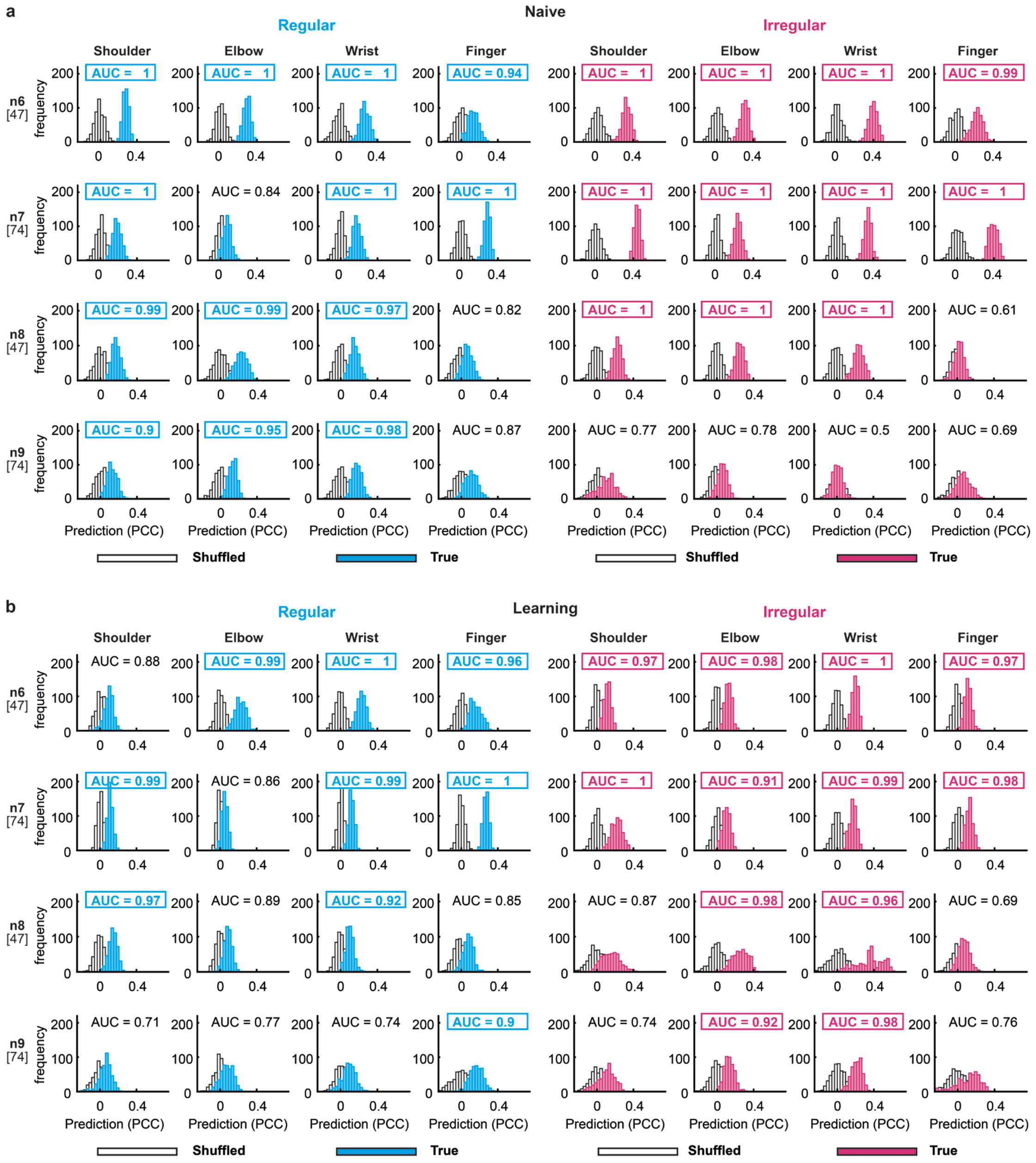

Supplementary Figure 3: True/shuffled encoding for naive and learning phase.
(a) Naive training phase: Prediction histograms: One 30% test set was randomly selected from 500 shuffled datasets (white for regular and irregular) or 500 30% test sets were randomly selected from the one true data set (cyan for regular, magenta for irregular). The 2 distributions of 500 predictions in each recorded neuronal network (n6-n9, number in square brackets below corresponds to the cell count of each neuronal network) during the naive phase are compared by calculating the area under the curve (AUC) of a ROC-analysis. AUC-values ≥ 0.9 in the respective neuronal network are regarded as significant encoding. **(b)** Same analyses for the learning phase. Note that high encoding in the shuffled distribution can arise as a consequence of the random shuffling and additionally by random selection of the test set. Still, true and shuffled distributions are in some cases completely separated.

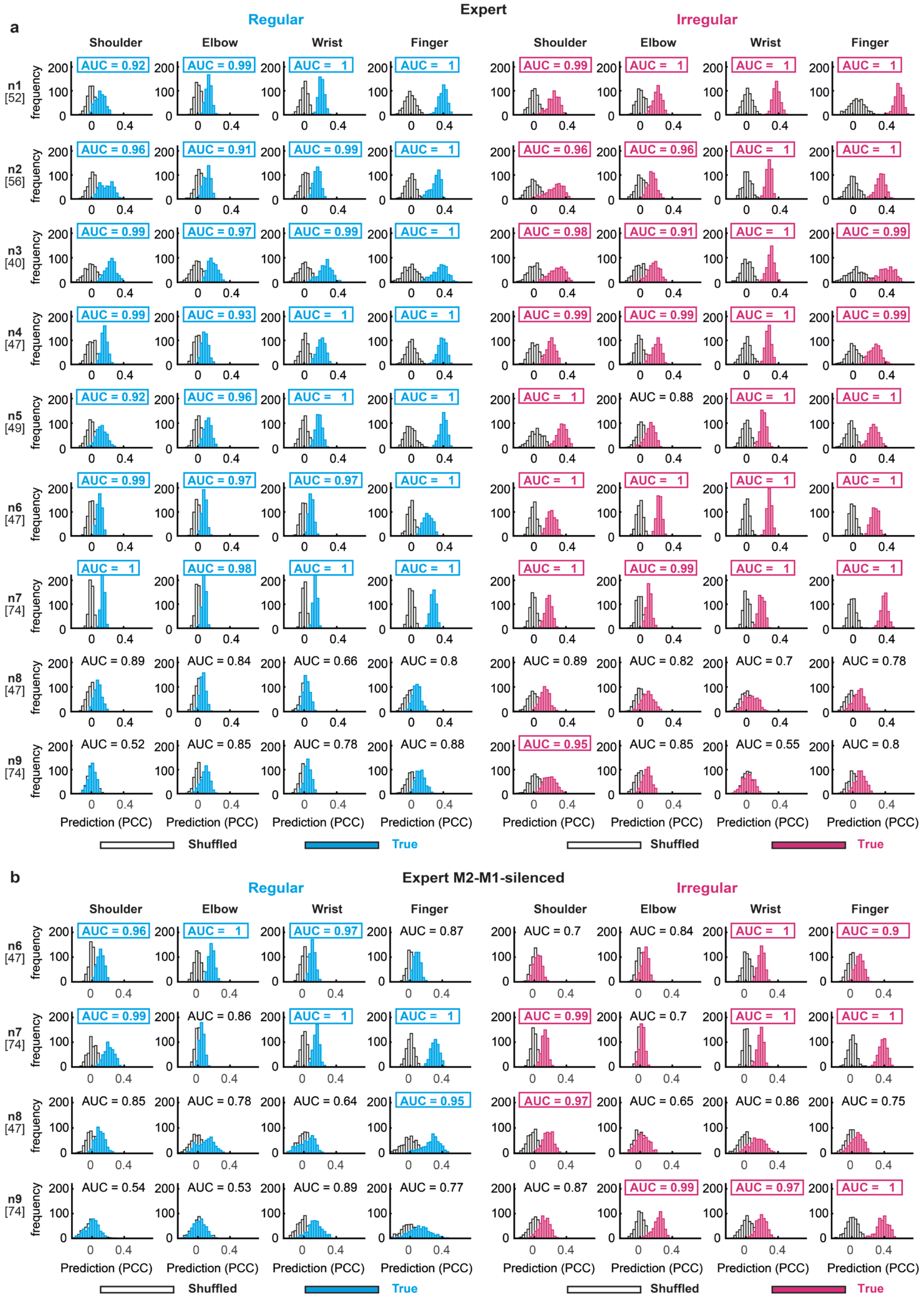

Supplementary Figure 4: True/shuffled encoding for expert phase and M2-M1-silencing. Same analysis procedure and conventions as in supplementary figure 3. **(a)** Encoding of forelimb joint movements in M1 L2/3 during the expert training phase. Altogether 9 neuronal networks (n1-n9, number in square brackets below corresponds to the cell count of each neuronal network) in M1 L2/3 have been recorded. **(b)** Encoding of forelimb joint movements in M1 L2/3 during chemogenetic silencing of M2-M1-projections using the DREADD-construct (expert M2-M1 silenced phase).

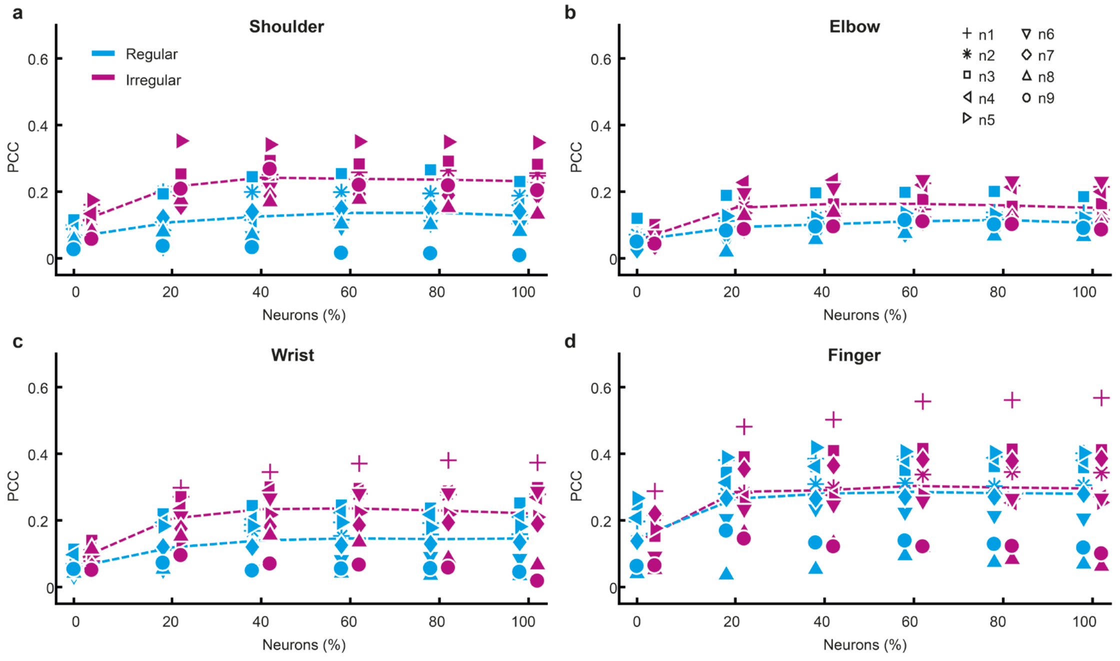

Supplementary Figure 5: Encoding of joint angles as a function of population size.
(**a**) Prediction of the shoulder joint angle as a function of population size, quantified by the Pearson correlation coefficient (PCC) between real and predicted joint angle traces; for each neuronal network n1-n9, cells with decreasing prediction strength have been added to the population coding, starting with best joint-angle-predictive single cell. Cyan symbols: Regular. Magenta symbols: Irregular. Dashed cyan curve: Mean for the regular condition. Dashed magenta curve: Mean for the irregular condition. (**b**), (**c**), and (**d**): Data for elbow, wrist and finger joints, respectively. Same conventions as in (a). Saturating prediction accuracy is mostly achieved after inclusion of 20-40% of the population size. Analysis refers to the expert phase.

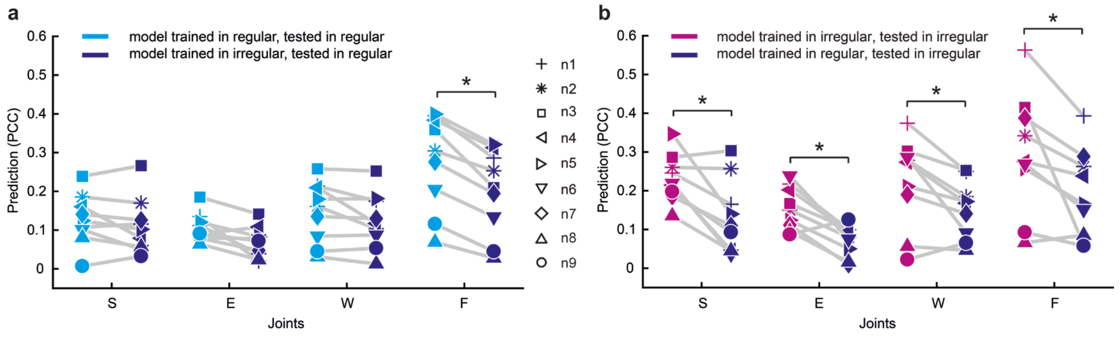

Supplementary Figure 6: Encoding with random forest trained in opposite condition. (**a**) Original decoding in the regular context (cyan) and decoding in the regular context if the prediction model was created from the same neuronal network in the irregular condition (purple; 70% training sets on the irregular condition, 30% test sets in the regular condition). Prediction power significantly decreases for finger movements. (**b**) Original decoding in the irregular context (magenta) and decoding in the irregular context if the prediction model was created from the same neuronal network in the regular condition (purple; 70% training sets in the regular condition, 30% test sets in the irregular condition). Prediction power significantly decreases for all joints. Asterisks indicate $P<0.05$ (paired t-test, P-value adjusted according to Holm-Bonferroni).

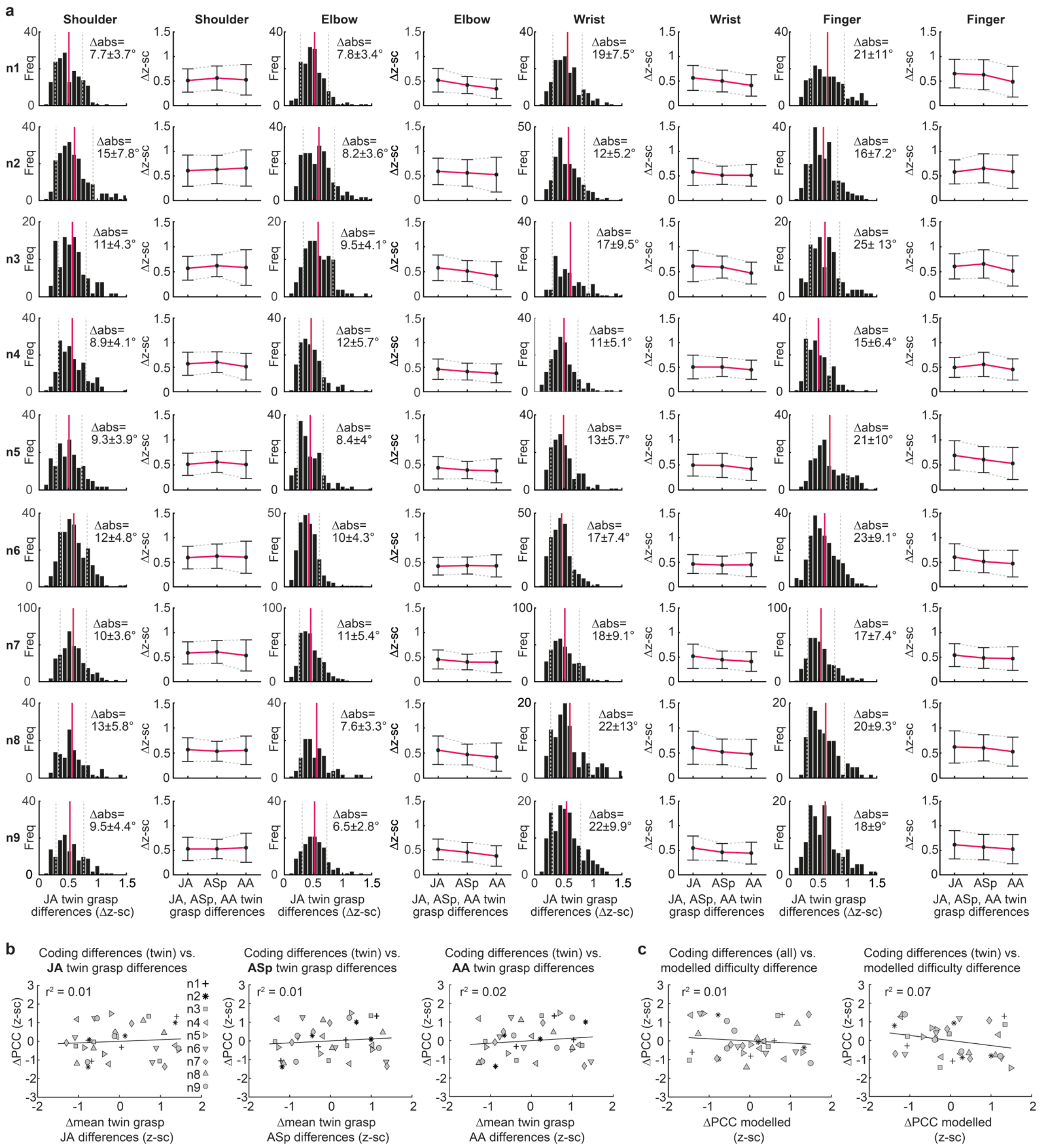

Supplementary Figure 7: Relationship of encoding with potential confounding factors. (**a**) Left panels: Histograms of joint angle (JA) differences between all twin grasps for regular and irregular. Distributions show differences of z-scored JAs, and respective mean (solid red line) and s.d. (dashed grey lines) are visible in each plot; mean and s.d. of twin grasp differences, when JAs were not z-scored, are shown as text inlays (Δabs); Freq = frequency. Right panels: Comparison between differences of z-scored JAs, z-scored joint angle speeds (ASp) and z-scored joint angle accelerations (AA), when twin grasps are regarded; for ΔJA, ΔASp and ΔAA, mean±s.d. is shown. Note that joint angles, their speeds and accelerations were controlled similarly for each joint by the twin grasp pruning. (**b**) Relationship of encoding differences (ΔPCC) with mean JA differences, mean ASp differences as well as mean AA differences for each joint during twin grasps. (**c**) Relationship between true encoding differences and modelled encoding differences when a single variable such as general task difficulty would have affected encoding differences between the regular and irregular condition similarly across joints, separately for the whole data set (left panel) and for twin grasps (right panel). Increased task difficulty was modelled by multiplying the baseline encoding on the regular wheel with arbitrary positive factors between 0 and 10 (the regression analysis is thereby not affected by the value of the respective factor). b, c: Linear regression with clustered standard error (robust), cluster variable = neuronal network).

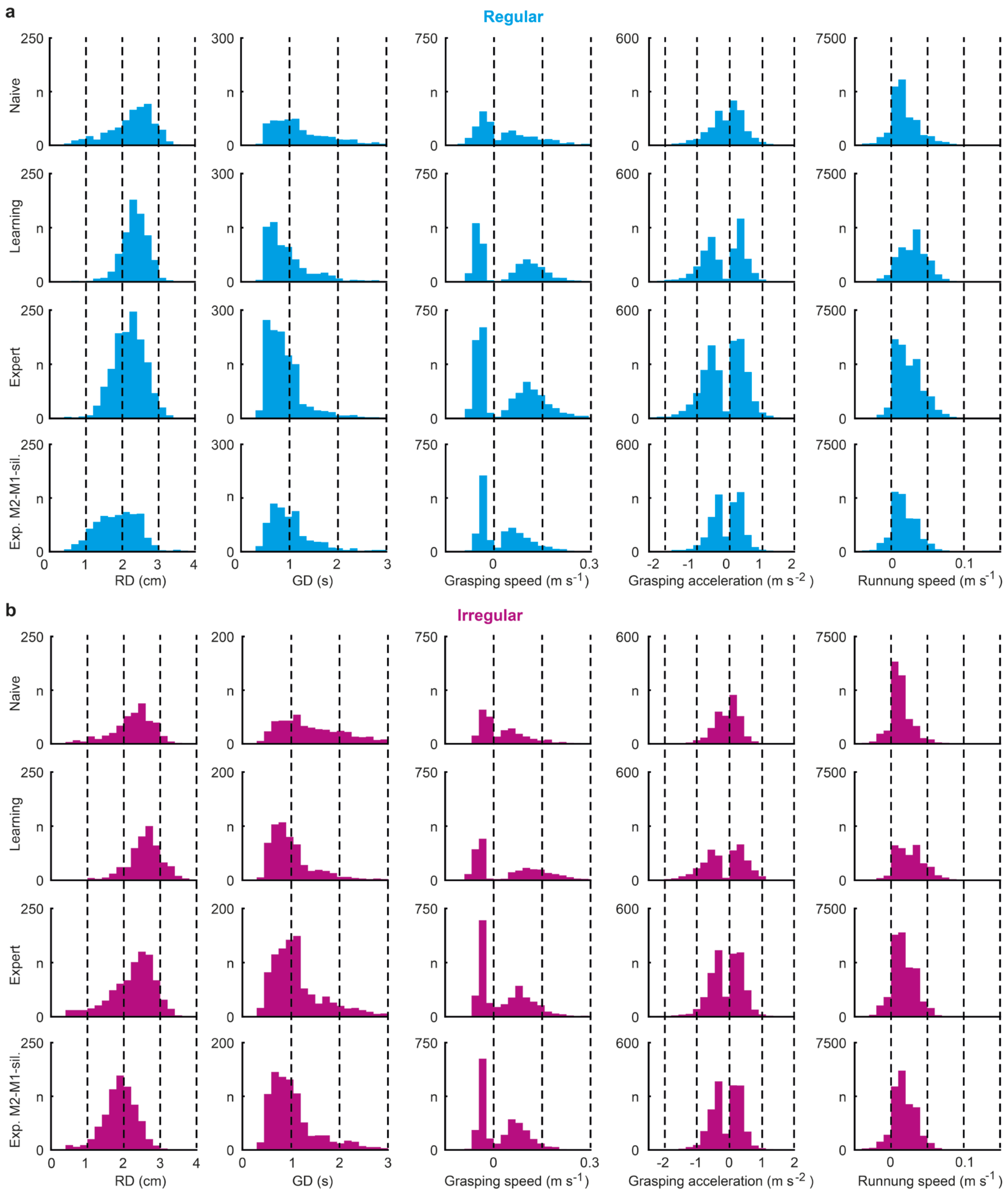

Supplementary Figure 8: Kinematic features during learning and M2-M1-silencing.
(a) Histograms of maximal reaching distance (RD) during each grasp, grasping duration (GD), mean grasping speed during paw reaching/retraction (positive/negative values), mean grasping acceleration during reaching/retraction (positive/negative values) and running speed during the three learning phases as well as during silencing of M2-M1-projections in the expert phase (Exp. M2-M1-sil.) when mice performed skilled locomotion on the regular wheel (cyan). Same conventions as in Fig. 1b,d. **(b)** Same analyses when mice performed skilled locomotion on the irregular wheel (magenta). Note that both across phases and conditions, the distributions of basic kinematic features are similar. Data pooled across animals of the second experimental series (m6 and m7).

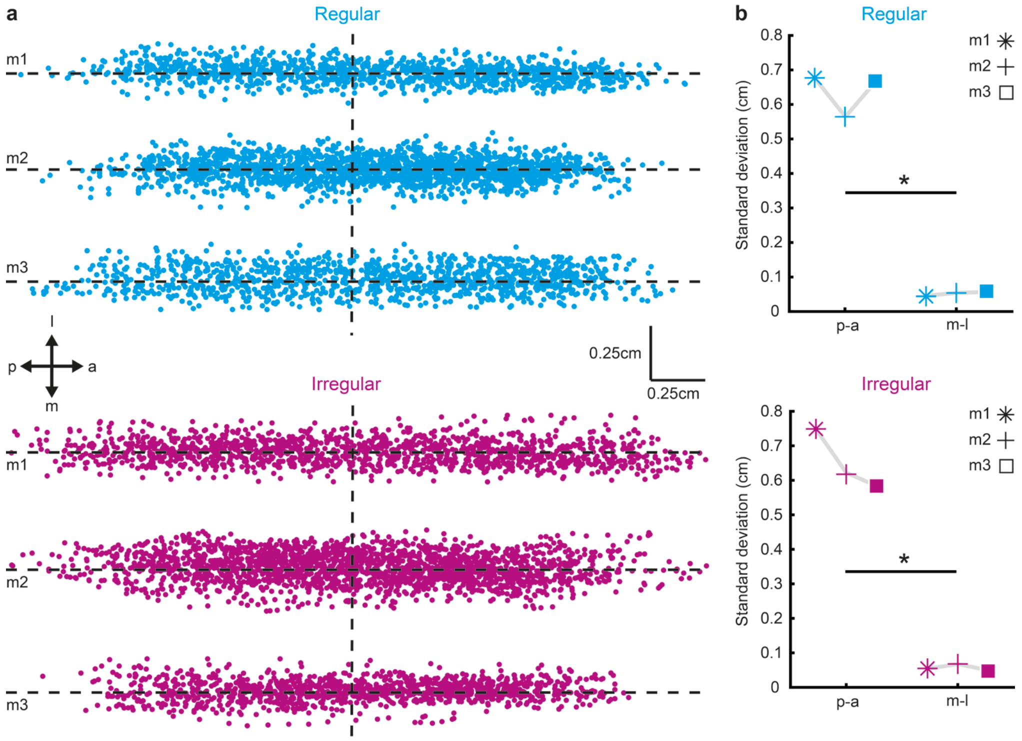

Supplementary Figure 9. Forepaw movement within the horizontal plane.
(a) In the first three animals, we recorded ten runs with a camera placed underneath the animals to track their forepaw movement within the horizontal plane. Both for the regular (cyan dots) and irregular (magenta dots) condition, forepaw movement in the medio-lateral direction (m-l) occurred to a negligible degree when compared to the posterior-anterior (p-a) direction. **(b)** During locomotion on the regular (cyan) and irregular (magenta) wheel, standard deviations of tracking dots in the m-l direction (0.04-0.07 cm) are significantly lower than in the p-a direction (0.56-0.75 cm). Asterisk indicates $P<0.01$; paired t-test; n = 3.

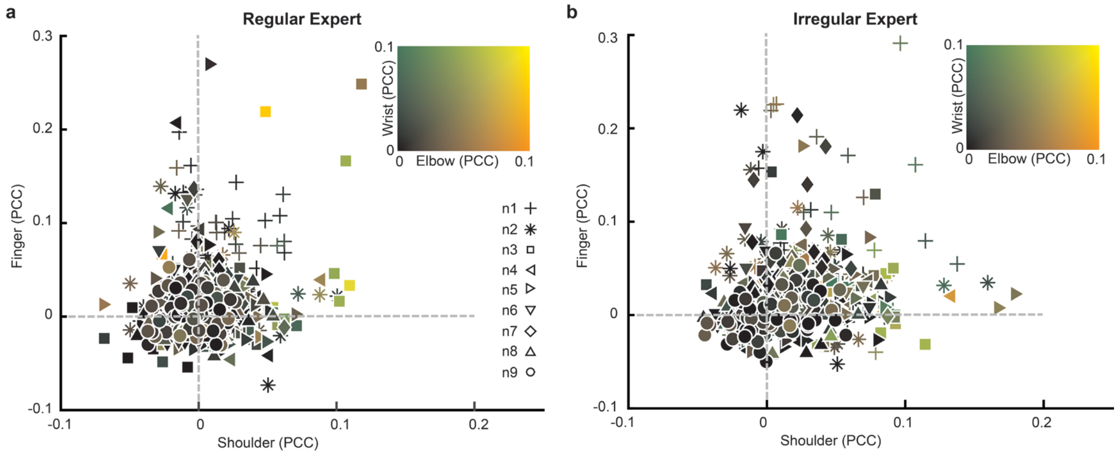

Supplementary Figure 10: Encoding of joint angles with activity of individual cells.
(**a**) Four-dimensional plot showing the prediction of forelimb joint angles from the activity of individual cells, based on the Pearson correlation coefficient (PCC) between real and predicted joint angle traces in the test sets of the cross-validation procedure. Prediction values from all neurons of the 9 recorded neuronal networks are shown for the regular pattern. For each cell, the prediction for the shoulder and finger angles can be concluded from the spatial location in the diagram while the prediction for elbow and wrist can be derived from the color code in the upper right corner. (**b**) same conventions as in (a) but for the irregular condition. Each symbol represents data of a different neuronal network; n1 – n9 refers to neuronal networks 1 to 9. Note that negative correlation means that the relationship of this neuron to the respective joint was opposite between training and test set. Therefore, the cross-validated correlation values of cells that significantly encode a joint in the complete dataset, are positive in this plot.

Supplementary Movie 1. Forelimb tattoo tracking during locomotion on ladder wheels. Grasping actions during skilled locomotion on the irregular wheel are shown, along with stick figure overlays (magenta) that depict the result of forelimb tattoo tracking. Permanent tattoos on scapula, shoulder, wrist, metacarpophalangeal joint and digit tip were tracked semi-automatically using the custom-made software ClickJoint. Due to larger skin movement in the elbow area, the elbow joint was calculated by assuming fixed shoulder-to-elbow and elbow-to-wrist distances.

Supplementary Movie 2. Classification of three different grasp types. Stick-figure movies of forelimb movements showing individual grasping actions (grey) along with the mean grasping action (green), separately for each of the three classified grasp types. To facilitate the illustration, each grasping action is normalized in time from start to end of the grasp. Data from one example mouse.

Supplementary Movie 3. Neuronal activity during locomotion on ladder wheels. Videos illustrating skilled locomotion (left panels) and simultaneously recorded time series of two-photon calcium imaging data for a L2/3 neuronal population in M1 (right panels). The upper row displays data acquired when the mouse was running on the regular wheel, the lower row when the same mouse was running on the irregular wheel. $\Delta R/R$ values were measured in the same neuronal network under the two conditions and are overlaid in red pseudocolor code. Data from one example mouse. Scale bars 50 μm. Frame rate 18 Hz. Replay 1.5x real time.

Supplementary Movie 4. Encoding of joint angles with neuronal population activity. The upper row displays data for the regular wheel, the lower panel for the irregular wheel. Left panels: Stick-figure movie of forelimb grasps showing the real movements of individual joints (grey) and the cross-validated prediction based on the combined activity of all neurons in the recorded network (purple) with the shoulder point affixed. Middle panels: Bar graph illustrating the temporal evolution of the Pearson’s correlation coefficients (PCC) between real and predicted joint angles accumulated over the time period shown. Right panels: Subplots showing the same grasping actions as in the left panels, but with each of the four joints affixed.
