# Supplementary material for: Context-dependent limb movement encoding in neuronal populations of motor cortex
Source: Nat Commun. 2019 Oct 23;10:4812. doi: 10.1038/s41467-019-12670-z (PMC6811620; doi:10.1038/s41467-019-12670-z)
Supplement: Supplementary file 2 — Reporting Summary [file 41467_2019_12670_MOESM2_ESM.pdf]

nature research

Corresponding author(s): Wolfgang Omlor
Fritjof Helmchen

Last updated by author(s): Aug 31, 2019

Reporting Summary

Nature Research wishes to improve the reproducibility of the work that we publish. This form provides structure for consistency and transparency in reporting. For further information on Nature Research policies, see Authors & Referees and the Editorial Policy Checklist.

Statistics

For all statistical analyses, confirm that the following items are present in the figure legend, table legend, main text, or Methods section.

n/a	Confirmed	
☐	☒	The exact sample size (*n*) for each experimental group/condition, given as a discrete number and unit of measurement
☐	☒	A statement on whether measurements were taken from distinct samples or whether the same sample was measured repeatedly
☐	☒	The statistical test(s) used AND whether they are one- or two-sided<br>*Only common tests should be described solely by name; describe more complex techniques in the Methods section.*
☒	☐	A description of all covariates tested
☐	☒	A description of any assumptions or corrections, such as tests of normality and adjustment for multiple comparisons
☐	☒	A full description of the statistical parameters including central tendency (e.g. means) or other basic estimates (e.g. regression coefficient) AND variation (e.g. standard deviation) or associated estimates of uncertainty (e.g. confidence intervals)
☐	☒	For null hypothesis testing, the test statistic (e.g. *F*, *t*, *r*) with confidence intervals, effect sizes, degrees of freedom and *P* value noted<br>*Give P values as exact values whenever suitable.*
☒	☐	For Bayesian analysis, information on the choice of priors and Markov chain Monte Carlo settings
☒	☐	For hierarchical and complex designs, identification of the appropriate level for tests and full reporting of outcomes
☐	☒	Estimates of effect sizes (e.g. Cohen's *d*, Pearson's *r*), indicating how they were calculated

Our web collection on statistics for biologists contains articles on many of the points above.

Software and code

Policy information about availability of computer code

Data collection	Behavior setups were programmed as a custom software written in LabVIEW 2011. Two-photon calcium imaging data was collected using HelioScan (based on LabView) and Scope (based on C++) software.
Data analysis	All data were analyzed in Matlab 2017a (Mathworks) using custom written scripts. The script library for data analysis will be available upon request.

For manuscripts utilizing custom algorithms or software that are central to the research but not yet described in published literature, software must be made available to editors/reviewers. We strongly encourage code deposition in a community repository (e.g. GitHub). See the Nature Research guidelines for submitting code & software for further information.

Data

Policy information about availability of data

All manuscripts must include a data availability statement. This statement should provide the following information, where applicable:

- Accession codes, unique identifiers, or web links for publicly available datasets
- A list of figures that have associated raw data
- A description of any restrictions on data availability

Data will be available upon reasonable request.

Field-specific reporting

Please select the one below that is the best fit for your research. If you are not sure, read the appropriate sections before making your selection.

☒ Life sciences ☐ Behavioural & social sciences ☐ Ecological, evolutionary & environmental sciences

Life sciences study design

All studies must disclose on these points even when the disclosure is negative.

Sample size	n = 9 for analyses regarding neuronal networks since 9 neuronal networks were recorded. n = 7 for analyses regarding behavior variables of animals since behavior variables came from 7 mice. The exact sample size of each analysis is given in the main text and/or figure. The sample size in this study is typical for studies in the field.
Data exclusions	No data was excluded.
Replication	For encoding of joint angles, the cross-validation procedure was used with 500 iterations for each neuronal network. The conclusions we made are based on the reproducibility of this analysis across all neuronal networks.
Randomization	The sequence of the regular and irregular condition was randomized between neuronal networks.
Blinding	Investigators were not blinded.

Reporting for specific materials, systems and methods

We require information from authors about some types of materials, experimental systems and methods used in many studies. Here, indicate whether each material, system or method listed is relevant to your study. If you are not sure if a list item applies to your research, read the appropriate section before selecting a response.

Materials & experimental systems

n/a	Involved in the study	
☒	☐	Antibodies
☒	☐	Eukaryotic cell lines
☒	☐	Palaeontology
☐	☒	Animals and other organisms
☒	☐	Human research participants
☒	☐	Clinical data

Methods

n/a	Involved in the study	
☒	☐	ChIP-seq
☒	☐	Flow cytometry
☒	☐	MRI-based neuroimaging

Animals and other organisms

Policy information about studies involving animals; ARRIVE guidelines recommended for reporting animal research

Laboratory animals	Adult (5-6 weeks) male transgenic ChR2 mice (Thy1-COP4/EYFP).
Wild animals	No wild animals were used.
Field-collected samples	The study did not include samples collected from the field.
Ethics oversight	All experimental procedures were carried out according to the guidelines of the Veterinary Office of Switzerland and approved by the Cantonal Veterinary Office in Zurich.

Note that full information on the approval of the study protocol must also be provided in the manuscript.
