# Supplementary material for: Context-dependent limb movement encoding in neuronal populations of motor cortex
Source: Nat Commun. 2019 Oct 23;10:4812. doi: 10.1038/s41467-019-12670-z (PMC6811620; doi:10.1038/s41467-019-12670-z)
Supplement: Supplementary file 3 — Description of Additional Supplementary Files [file 41467_2019_12670_MOESM3_ESM.pdf]

Description of Additional Supplementary Files

Supplementary Movie 1.

Forelimb tattoo tracking during locomotion on ladder wheels. Grasping actions during skilled locomotion on the irregular wheel are shown, along with stick figure overlays (magenta) that depict the result of forelimb tattoo tracking. Permanent tattoos on scapula, shoulder, wrist, metacarpophalangeal joint and digit tip were tracked semiautomatically using the custom-made software ClickJoint. Due to larger skin movement in the elbow area, the elbow joint was calculated by assuming fixed shoulder-to-elbow and elbow-towrist distances.

Supplementary Movie 2.

Classification of three different grasp types. Stick-figure movies of forelimb movements showing individual grasping actions (grey) along with the mean grasping action (green), separately for each of the three classified grasp types. To facilitate the illustration, each grasping action is normalized in time from start to end of the grasp. Data from one example mouse.

Supplementary Movie 3.

Neuronal activity during locomotion on ladder wheels. Videos illustrating skilled locomotion (left panels) and simultaneously recorded time series of twophoton calcium imaging data for a L2/3 neuronal population in M1 (right panels). The upper row displays data acquired when the mouse was running on the regular wheel, the lower row when the same mouse was running on the irregular wheel. ΔR/R values were measured in the same neuronal network under the two conditions and are overlaid in red pseudocolor code. Data from one example mouse. Scale bars 50 µm. Frame rate 18 Hz. Replay 1.5x real time.

Supplementary Movie 4.

Encoding of joint angles with neuronal population activity. The upper row displays data for the regular wheel, the lower panel for the irregular wheel. Left panels: Stick-figure movie of forelimb grasps showing the real movements of individual joints (grey) and the cross-validated prediction based on the combined activity of all neurons in the recorded network (purple) with the shoulder point affixed. Middle panels: Bar graph illustrating the temporal evolution of the Pearson's correlation coefficients (PCC) between real and predicted joint angles accumulated over the time period shown. Right panels: Subplots showing the same grasping actions as in the left panels, but with each of the four joints affixed.
